# Analysis on the Clinical and Pathological Features and Prognosis of Familial Gastric Cancer in South China Population: A Single-Center Study of 724 Patients

**DOI:** 10.1155/2012/641218

**Published:** 2012-11-07

**Authors:** Jun Lu, Chang-Ming Huang, Chao-Hui Zheng, Ping Li, Jian-Wei Xie, Jia-Bin Wang, Jian-Xian Lin

**Affiliations:** Department of Gastric Surgery, Union Hospital, Fujian Medical University, Fuzhou 350001, China

## Abstract

Gastric cancer is the second most common cause of cancer death worldwide. It is estimated that 5–10% of gastric cancer cases have a familial association; however, knowledge concerning the clinical, pathological features and prognosis to familial gastric cancer is currently limited. To our best knowledge, this is the largest number of single center patients reported in southern China. Our research can help these rare families to obtain optimal treatment in the future. Our work is supported by Union Hospital of Fujian Medical University.

## 1. Introduction

Gastric cancer is still one of the most frequent causes of cancer-related deaths. Although its incidence has decreased in recent years, it is still high in Eastern Asia, including China. Familial gastric cancer (FGC) has a lower incidence in Western countries, only 1%–3% of patients have been diagnosed as family gastric cancer [1]. The incidence of FGC in China is higher than that in the West. Reports indicate that in northern China about 7.8% of patients with gastric cancer can be diagnosed as FGC [2]. Hereditary Diffuse Gastric Cancer (HDGC) is the only familial cancer syndrome which primarily affects the stomach and for which a mutation has been identified. Asymptomatic family members have to make a choice about whether to have genetic testing and individuals who test positive for an inherited E-cadherin mutation have to make difficult decisions about whether to option for endoscopic surveillance or prophylactic gastrectomy. However, it should be remembered that mutations of the E-cadherin gene (CDHI) only in one-third of familial gastric cancer cases are only relevant for diffuse-type gastric cancer, and the observed mutations were different in Western and Asian ethic groups [3]. At present, most scholars are focusing on the level of familial gastric cancer gene pathogenesis [3–6], but the reports on analysis of clinical and pathological features and prognosis are rare. This study retrospectively analyzed 51 cases of familial gastric cancer (FGC) in patients with clinical and pathological data and prognosis, treated by the Department of Gastric Surgery, Union Hospital of Fujian Medical University from January 2004 to December 2006 and compared with the 673 cases of sporadic gastric cancer (SGC) within the same period. This is so far the first reported clinical and pathological features and prognosis of patients with familial gastric cancer in southeast China population aimed at improving the diagnosis and treatment of familial gastric cancer.

## 2. Materials and Methods

### 2.1. Patients

The inclusion criteria for FGC are as follows: (1) first and (or) second degree relatives have two cases or more than two cases in any tissue type of gastric cancer, one confirmed before 50 years; (2) first and (or) second degree relatives have three or more than three cases of gastric cancer, with no age limited. Hereditary nonpolyposis colorectal cancer (HNPCC), family gonadal fibromatosis (FAP), Li-Fraumeni syndrome, Cowden syndrome, and Peutz-Jegher syndrome were excluded [6]. Those that do not comply with the above standards of familial gastric cancer are defined as sporadic gastric cancer (SGC). This group has collected the clinical data of the 724 patients with gastric cancer who accepted the radical surgical treatment, cured by the Department of Gastric Surgery, Union Hospital of Fujian Medical University from January 2004 to December 2006, among which there are 51 cases of FGC and 673 cases of SGC, accounting for 7.0% and 93.0% of the total number of patients with gastric cancer during the period, respectively. The comparison of general information of patients in two groups is shown in Table 1.

### 2.2. Postoperation Follow-Up Methods

Patients were followed up by hand, using the outpatient door visit, letter, and telephone followup. The survival time was the time from diagnosis until the last contact, the date of death, or the date that the survival information was collected. In addition to the patients who died, all surviving patients were followed up for more than five years.

### 2.3. Statistical Processing

Statistical analysis and graphics were performed by SPSS 17.0 statistical software package. The measurement data were compared with Chi-square test. The survival rate was calculated according to the Kaplan-Meier method, and the comparison of the survival rate was tested using the Log-rank method. Cox proportional hazards model was adopted for multivariate analysis of prognosis. It is considered statistically significant when *P* values are <0.05.

## 3. Results

### 3.1. Comparison of the Clinical and Pathological Data between Patients with FGC and Patients with SGC

The comparison showed that the proportion of patients with FGC under the age of 50 was significantly higher than the SGC group, but in terms of the tumor site, tumor size, histological type, depth of invasion, lymph node metastasis, the two groups have no statistical difference (Table 1).

### 3.2. Comparison of the Prognosis between FGC and SGC

**Figure 1:**
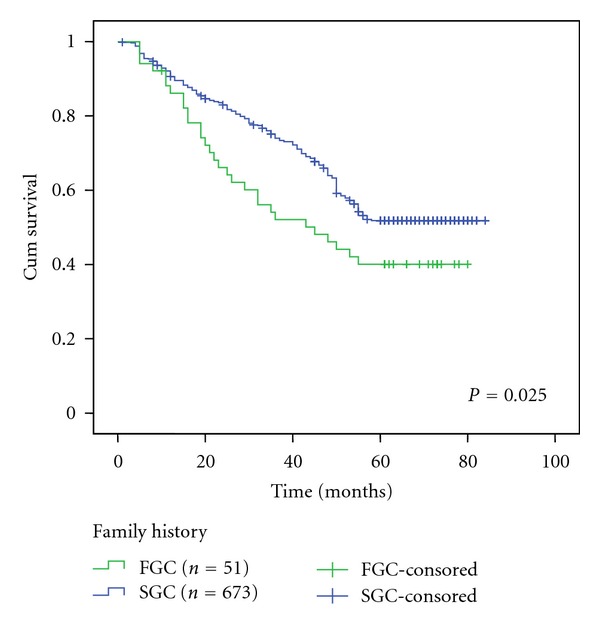


661 cases (91.3%) of patients were followed up for 1–84 months. The postoperative 5-year survival rates in FGC and SGC patients were 40.1% and 51.8%, respectively. The difference was statistically significant (*P* < 0.05, Figure 1).

### 3.3. Univariate Analysis of Patients with FGC and Patients with SGC

The univariate analysis found that the factors that affect FGC prognosis are lymph node metastasis, depth of invasion, tissue type, and tumor size, while the factors that impact the prognosis of the SGC in patients are lymph node metastasis, depth of invasion, histological type, tumor size, and tumor location (*P* < 0.05, Table 2).

### 3.4. Multivariate Analysis of Patients with FGC and Patients with SGC

Cox proportional hazards model analysis showed that lymph node metastasis and depth of invasion are the independent factors to affect the prognosis of FGC patients; lymph node metastasis, depth of invasion, and tumor size are the independent factors affecting SGC prognosis (*P* < 0.05, Table 3).

### 3.5. Comparison between Patients with FGC and Patients with SGC in Different T Stages

**Figure 2:**
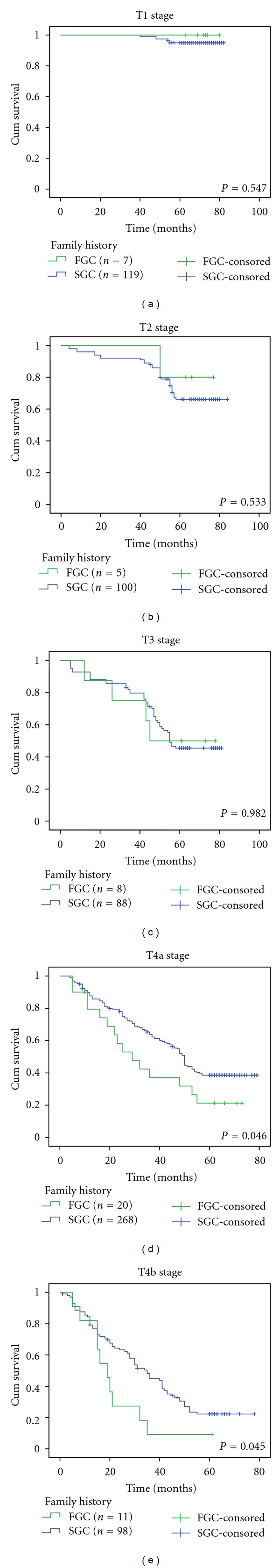


Depth of invasion stratified analysis found that the postoperative 5-year survival rates of FGC and SGC patients in stages T1, T2, and T3 and were 100% and 94.9%, 80.0% and 66.1%, and 50.0% and 45.5%, respectively, and the differences were not statistically significant (*P* > 0.05, Figures 2(a)–2(c)). The postoperative 5-year survival rates of FGC and SGC patients in stages T4a, T4b 21.2% and 38.6%, and 9.1% and 22.3%, and the differences were statistically significant (*P* < 0.05, Figures 2(d) and 2(e)).

### 3.6. Comparison of Prognosis between Patients with FGC and Patients with SGC in Different N Stages

**Figure 3:**
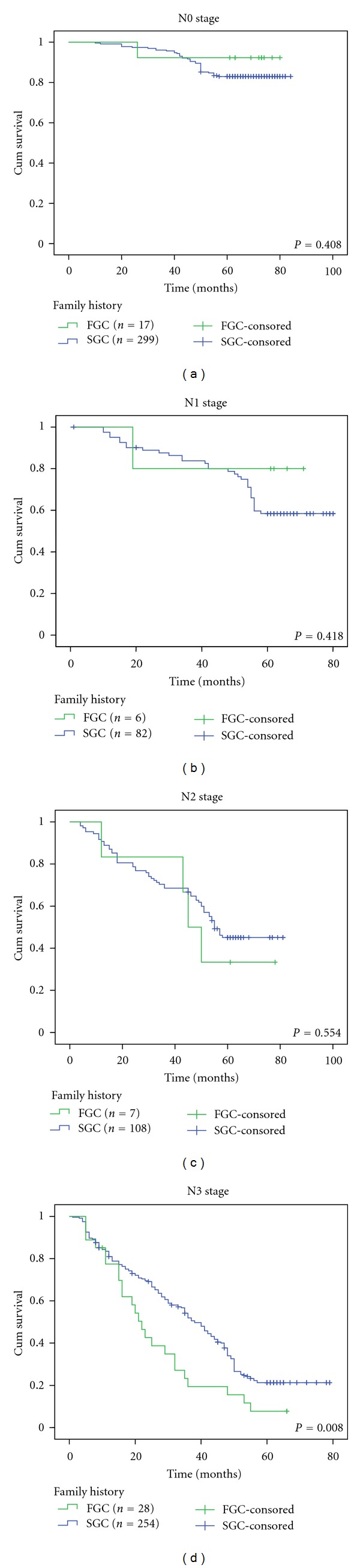


Stratified analysis of lymph node metastasis showed that the post-operative 5-year survival rates of FGC and SGC patients in stages N0, N1, and N2 were 92.3% and 83.0%, 80.0% and 58.4%, and 33.3% and 45.1%, respectively, and the differences were not statistically significance (*P* > 0.05, Figures 3(a)–3(c)). The postoperative 5-year survival rates of FGC and SGC patients in stage N3 were 7.7% and 21.3%, respectively, and the difference was statistically significant (*P* < 0.05, Figure 3(d)).

## 4. Discussion 

Familial gastric cancer (FGC) is regarded as an autosomal dominant tumor syndrome. The characteristics of the sick people include the apparently younger ages and familial aggregation [7, 8]. According to the Lauren pathological type, FGC defined in this study can be divided into two categories: hereditary diffuse gastric cancer (HDGC) and familial intestinal gastric cancer (FIGC) [9]. In 1998, Guilford and his colleagues first discovered that the HDGC is connected with the E-cadherin gene (CDH1) mutations, which opened the prelude of the the FGC genetics research [10]. So far, although there were a large number of studies indicating that the occurrence of HDGC is closely related to CDH1 gene [3, 4], still the pathogenesis remains unclear [11]. The research made by Yamada et al. [12] revealed that CDH1 gene mutation rate in Japanese FGC patients was 15.4% and that the incidence of FGC was connected with the environmental factors, such as smoking, Helicobacter pylori infection, high-salt diet, or other genes (e.g., p53, the MET, STK11) mutations.

It has been reported that FGC usually has a unique biological behavior [13, 14]: early onset, poor tumor differentiation, and the trend of parenteral tumors and multiple primary cancers. As for the distribution of tumor location, Charlton et al. [15] reported that hereditary diffuse gastric lesions are mainly in distal gastric cancer and Rogers et al. [16] reported that early lesions are often multifocal, whose location is more common in the proximal stomach. Yan-Wei et al. [17] reviewed 81 cases of FGC patients and indicated that the age of onset, depth of invasion, lymph node metastasis, pathological stage, and other aspects in FGC and SGC patients were not statistically different. Our data showed that, compared with the SGC, the FGC patients have earlier age of onset, but the tumor site, tumor size, histological type, depth of invasion, and lymph node metastasis were not statistically different.

Despite the detailed description of the pathogenesis of the FGC gene level by many scholars and the gradual discovery of its clinical and pathological features, its low incidence makes the postoperative long-term efficacy still poorly recognized. In recent years, some scholars believe that prophylactic total gastrectomy on patients whose families have a gastric cancer history and who are detected CDH1 gene mutation can help to improve their prognosis [18, 19]. Yan-Wei et al. [17] study revealed that the postoperative 5-year survival rates of FGC and SGC patients who underwent radical surgery were 48% and 57%, and the difference was statistically significant (*P* < 0.05); P53-positive and AJCC staging are the independent factors impacting FGC prognosis. In our study, 5-year survival rate in patients with FGC was obviously worse than that in SGC patients (*P* < 0.05). Further prognosis stratification analysis of the depth of invasion and lymph node metastasis indicated that the FGC patients in Stages T1–3 or N0–2 who underwent radical surgery can achieve similar prognosis to SGC. Therefore, we believe that early diagnosis and treatment of FGC is critical; timely and radical surgery can improve the prognosis of patients. In addition, we also found that the FGC and SGC prognostic factors are not consistent, and FGC may be a special type of gastric cancer.

In summary, this study showed that FGC has early onset; the lymph node metastasis and depth of invasion are the independent prognostic factors. FGC patients in Stage T1–3 or N0–2 who underwent radical surgery can achieve similar prognosis to SGC; however, patients in Stages T4 or N3 have poorer prognosis. We believe that, the key to improve the prognosis of FGC patients is early diagnosis and treatment. Besides, a further analysis with a larger sample is extremely essential to verify the findings in our study.

## Figures and Tables

**Table 1:** The contrast of clinicopathological parameters between patients with familial gastric cancer (FGC) and patients with sporadic gastric cancer (SGC) (*n*, %).

	FGC	SGC	*P* value
*n*	51	673	
Age (y)			0.001
≤50	26 (51.0)	193 (28.7)	
>50	25 (49.0)	480 (71.3)	
Gender			0.510
Male	36 (70.6)	502 (74.6)	
Female	15 (29.4)	171 (25.4)	
Tumor size (cm)			0.052
≤5	24 (47.1)	415 (61.7)	
>5	27 (52.9)	258 (38.3)	
Tumor location			0.235
Upper	8 (15.7)	126 (18.7)	
Middle	6 (11.8)	113 (16.8)	
Lower	19 (37.3)	280 (41.6)	
Diffuse	18 (35.2)	154 (22.9)	
Pathology			0.919
Differentiated	21 (41.2)	282 (41.9)	
Undifferentiated	30 (58.8)	391 (58.1)	
Depth of invasion			0.549
T1	7 (13.7)	119 (17.7)	
T2	5 (9.8)	100 (14.9)	
T3	8 (15.7)	88 (13.1)	
T4a	20 (39.2)	268 (39.8)	
T4b	11 (21.6)	98 (14.6)	
Lymph node metastasis			0.201
N0	17 (29.3)	229 (34.0)	
N1	6 (10.3)	82 (12.2)	
N2	7 (12.1)	108 (16.0)	
N3	28 (48.3)	254 (37.8)	

**Table 2:** Univariate analysis of patients with familial gastric cancer (FGC) and patients with sporadic gastric cancer (SGC) by kaplan-meier method.

Characteristics	FGC			SGC		
	*n*	5-year survival rate (%)	*P* value	*n*	5-year survival rate (%)	*P* value
Age (y)			0.887			0.564
≤50	26	40.2		192	54.0	
>50	25	40.0		480	50.9	
Gender			0.947			0.082
Male	36	40.1		502	54.2	
Female	15	40.0		171	44.7	
Tumor size (cm)			0.017			0.000
≤5	24	56.7		415	67.3	
>5	27	25.9		258	25.0	
Tumor location			0.241			0.000
Upper	8	37.5		126	48.6	
Middle	6	83.3		113	65.6	
Lower	19	39.1		280	59.2	
Diffuse	18	27.8		154	30.0	
Pathology			0.008			0.000
Differentiated	21	61.9		282	68.3	
Undifferentiated	30	24.3		391	40.8	
Depth of invasion			0.000			0.000
T1	7	100.0		119	94.9	
T2	5	80.0		100	66.1	
T3	8	50.0		88	45.5	
T4a	20	21.2		268	38.6	
T4b	11	9.1		98	22.3	
Lymph node metastasis			0.000			0.000
N0	13	92.3		229	83.0	
N1	5	80.0		82	58.4	
N2	6	33.3		108	45.1	
N3	27	7.7		254	21.3	

**Table 3:** Independent prognostic factors at multivariate analysis by cox model.

Characteristics	*B*	SE	Wald	*P* value	RR	95% CI
FGC						
Lymph node metastasis	0.819	0.285	8.238	0.004	2.269	1.297–3.970
Depth of invasion	0.653	0.284	5.265	0.022	1.921	1.100–3.355
Pathology	0.134	0.427	0.099	0.753	1.144	0.495–2.640
Tumor size	0.327	0.427	0.587	0.444	0.721	0.312–1.665
SGC						
Lymph node metastasis	0.451	0.060	56.086	0.000	1.570	1.395–1.767
Depth of invasion	0.314	0.062	25.796	0.000	1.369	1.213–1.546
Pathology	0.249	0.130	3.693	0.055	1.283	0.995–1.645
Tumor size	0.553	0.126	19.186	0.000	1.738	1.357–2.226
Tumor location	0.042	0.040	1.095	0.295	1.043	0.964–1.127

## References

[1] Corso G, Marrelli D, Roviello F (2011). Familial gastric cancer: update for practice management. *Familial Cancer*.

[2] Wang B, Li Z, Liu C, Xu H, Jin F, Lu P (2010). Family history of cancer in Chinese gastric cancer patients. *Chinese-German Journal of Clinical Oncology*.

[3] Oliveira C, Seruca R, Carneiro F (2006). Genetics, pathology, and clinics of familial gastric cancer. *International Journal of Surgical Pathology*.

[4] Humar B, Guilford P (2009). Hereditary diffuse gastric cancer: a manifestation of lost cell polarity. *Cancer Science*.

[5] Guilford P, Humar B, Blair V (2010). Hereditary diffuse gastric cancer: translation of CDH1 germline mutations into clinical practice. *Gastric Cancer*.

[6] Park JG, Yang HK, Kim WH, Caldas C, Yokota J, Guilford PJ (2000). Report on the first meeting of the International Collaborative Group on Hereditary Gastric Cancer. *Journal of the National Cancer Institute*.

[7] Caldas C, Carneiro F, Lynch HAT (1999). Familial gastric cancer: overview and guidelines for management. *Journal of Medical Genetics*.

[8] Ebert MPA, Malfertheiner P (2002). Review article: pathogenesis of sporadic and familial gastric cancer—implications for clinical management and cancer prevention. *Alimentary Pharmacology and Therapeutics*.

[9] Frank TS (2001). Hereditary cancer syndromes. *Archives of Pathology & Laboratory Medicine*.

[10] Guilford P, Hopkins J, Harraway J (1998). E-cadherin germline mutations in familial gastric cancer. *Nature*.

[11] Barber M, Fitzgerald RC, Caldas C (2006). Familial gastric cancer—aetiology and pathogenesis. *Best Practice & Research*.

[12] Yamada H, Shinmura K, Ito H (2011). Germline alterations in the CDH1 gene in familial gastric cancer in the Japanese population. *Cancer Science*.

[13] Shinmura K, Kohno T, Takahashi M (1999). Familial gastric cancer: clinicopathological characteristics. RER phenotype and geIInIine p53 and E-cadherin mutations. *Carcinogenesis*.

[14] Kaurah P, MacMillan A, Boyd N (2007). Founder and recurrent CDH1 mutations in families with hereditary diffuse gastric cancer. *Journal of the American Medical Association*.

[15] Charlton A, Blair V, Shaw D, Parry S, Guilford P, Martin IG (2004). Hereditary diffuse gastric cancer: predominance of multiple foci of signet ring cell carcinoma in distal stomach and transitional zone. *Gut*.

[16] Rogers WM, Dobo E, Norton JA (2008). Risk-reducing total Gastrectomy for germline mutations in E-cadherin (CDH1): pathologic findings with clinical implications. *American Journal of Surgical Pathology*.

[17] Yan-Wei YE, Rui-Zheng D, Ye Z (2011). Prognostic analysis of familial gastric cancer in Chinese population. *Journal of Surgical Oncology*.

[18] Lewis FR, Mellinger JD, Hayashi A (2001). Prophylactic total gastrectomy for familial gastric cancer. *Surgery*.

[19] Chun YS, Lindor N, Smyrk TC (2001). Germline E-Cadherin gene mutations. Is prophylactic total gastrectomy indicated?. *Cancer*.

